# Validation of the Revised Stressful Life Event Questionnaire Using a Hybrid Model of Genetic Algorithm and Artificial Neural Networks

**DOI:** 10.1155/2013/601640

**Published:** 2013-02-07

**Authors:** Rasoul Sali, Hamidreza Roohafza, Masoumeh Sadeghi, Elham Andalib, Hassan Shavandi, Nizal Sarrafzadegan

**Affiliations:** ^1^Industrial Engineering Department, Sharif University of Technology, P.O. Box 11365-9466, Tehran, Iran; ^2^Mental Health Unit, Cardiovascular Research Center, Isfahan Cardiovascular Research Institute, Isfahan University of Medical Sciences, P.O. Box 81465-1148, Isfahan, Iran; ^3^Cardiac Rehabilitation Research Center, Isfahan Cardiovascular Research Institute, Isfahan University of Medical Sciences, Isfahan, Iran; ^4^Cardiovascular Research Center, Isfahan Cardiovascular Research Institute, Isfahan University of Medical Sciences, Isfahan, Iran

## Abstract

*Objectives*. Stressors have a serious role in precipitating mental and somatic disorders and are an interesting subject for many clinical and community-based studies. Hence, the proper and accurate measurement of them is very important. We revised the stressful life event (SLE) questionnaire by adding weights to the events in order to measure and determine a cut point. *Methods*. A total of 4569 adults aged between 18 and 85 years completed the SLE questionnaire and the general health questionnaire-12 (GHQ-12). A hybrid model of genetic algorithm (GA) and artificial neural networks (ANNs) was applied to extract the relation between the stressful life events (evaluated by a 6-point Likert scale) and the GHQ score as a response variable. In this model, GA is used in order to set some parameter of ANN for achieving more accurate results. *Results*. For each stressful life event, the number is defined as weight. Among all stressful life events, death of parents, spouse, or siblings is the most important and impactful stressor in the studied population. Sensitivity of 83% and specificity of 81% were obtained for the cut point 100. *Conclusion*. The SLE-revised (SLE-R) questionnaire despite simplicity is a high-performance screening tool for investigating the stress level of life events and its management in both community and primary care settings. The SLE-R questionnaire is user-friendly and easy to be self-administered. This questionnaire allows the individuals to be aware of their own health status.

## 1. Introduction

Importance of stressors and their impact on human life have attracted many researchers' interest in the recent years. When investigating stressors, their frequency and intensity are the most important characteristics that must be considered [1]. Not all stressors have the same impact. Some of them have more intense impact on the individual's life and some have less. The importance of the same stressor can also be different in various societies and cultures. Evaluation of the importance and impact of stressors is a very attractive subject in psychological studies and many studies have been performed in this field [2].

 To measure stressors, different scales and tools have been developed in developed countries [2–4]. Recently, the developing and use of stress measurement tools has also been the subject of many studies in developing countries as well [5].

The use of self-administered questionnaires is demonstrated to be a great tool to obtain useful information about the health status in epidemiologic studies and health surveys [6, 7]. Meaningful estimates of disease status could be obtained by cross-check and agreement between questionnaire data and standard criterion such as medical records. Many studies have been considered an agreement between questionnaire data and a criterion standard [7–13]. In these studies, researchers used user-friendly and easy to be self-administered questionnaires.

Being user-friendly for questionnaire provides a key characteristic and a better chance for success in questionnaire-based researches. One of the advantages of the user-friendly questionnaire is that individuals could become aware of their health status while using them.

Questionnaires should be designed in a way to be an indicator of health status or other under-research conditions. In other words, a user friendly questionnaire could easily provide information about the individual health status by comparing the scores that are calculated with its cut point.

By using machine learning algorithms such as artificial neural networks (ANNs) and determining a cut point we aimed to provide a weight for stressful life events that are introduced in the stressful life event (SLE) questionnaire. This way, a revised SLE questionnaire could be used as a screening tool to differentiate healthy individuals from those who are at risk of a disease.

## 2. Materials and Methods

### 2.1. Dataset

The data we used in this study is a part of the data collected for the Isfahan Healthy Heart Program (IHHP) research. IHHP is a community-based intervention program to prevent and control noncommunicable diseases. Details of the methodology used in IHHP including sampling strategies, survey instruments, data entry, and analysis in addition to the evaluation and followup of the subjects are described by Sarraf–Zadegan et al. elsewhere [14, 15].

In summary, IHHP includes a baseline survey, four follow-ups and eventually a final phase. The baseline survey was conducted in 2001 in 2 interventional and 1 referral regions. The interventional regions include Isfahan and Najafabad. We selected Arak as the referral region. Four phases of annual followup and evaluation were performed on independent samples from 2002 to 2005. The Final phase was conducted in 2007. According to the regional population distribution based on the CINDI protocol, a multistage cluster random sampling was applied in order to differentiate urban versus rural areas [14].

Data used for the current study include the information from 4569 adults aged over 18 years who have completed the final phase and have all their related data available. The related data include demographic information such as age, sex, and educational years. We asked all participants to complete the SLE questionnaire and the general health questionnaire-12 (GHQ-12). An informed consent was obtained from all subjects.

We evaluated the frequency and intensity of the stressful life events for participants by the SLE questionnaire which was developed and validated by Roohafza et al. [1]. This questionnaire consists of eleven domains identified by factor analysis, including home life, financial problems, social relation, personal conflict, job conflict, educational concern, job security, loss and separation, sexual life, daily life, and health concern. In each domain few questions with regard to the related stressful event exist. To score each stressful life event, we used a 6-point Likert scale (0 = never, 1 = very mild, 2 = mild, 3 = moderate, 4 = severe, and 5 = very severe). For SLE, the standardized Cronbach's alpha is 92%. 

The GHQ-12 was used for the validation and evaluation of the SLE questionnaire. The GHQ-12 is a well-established screening tool to detect psychological morbidity in community and clinical settings such as in primary care or general practice [16, 17]. It includes 12 items that are classified in two main areas such as the ability to carry out normal functions and the appearance of new and distressing experience. If the GHQ score is ≥4, the individual has a high stress level.

### 2.2. The Basic Concept of Artificial Neural Networks

**Figure 1:**
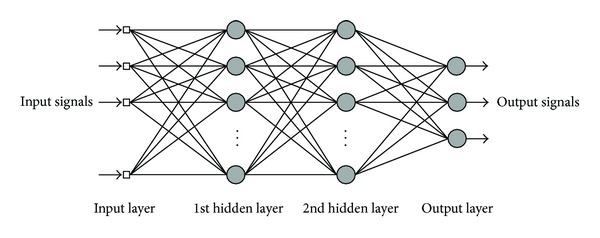
An example of an ANN with an input layer, 2 hidden layers, and an output layer.

Inspired from the biology of human brain, an artificial neural network (ANN) is a parallel processing system which has an executive performance [18]. These systems are able to learn rules embedded in the data, and due to this ability, it is usually used as a model for complex relationships between inputs and outputs or to find patterns in data. Pattern recognition is achieved by adjusting the ANN parameters by an error reduction method through learning. ANN consists of one input layer, one output layer, and one or more hidden layers.  Figure 1 demonstrates an example of an ANN with an input layer, 2 hidden layers, and an output layer.

In Figure 1, circles in layers show neurons. The input layer takes input signals and transfers them to the hidden layers. The output layer takes the outgoing signal from the last hidden layer and presents the appropriate result.

Each layer is determined by its weight matrix. The matrix multiplication operation of the various layers along with applying transfer functions is formed network structure and map input signals transfer to output data. If the network consists of *n* layers, the estimated mapping function *F* will be

(1) F(X)=ψn(Wn,n−1ψn−1(Wn−1,n−2ψn−2(⋯ψ1(IW1,1·X)⋯))),

where *X*: input signals vector, *n*: total number of layers, *ψ*_*i*_(·): transfer function layer *i*, *W*_*i*,*j*_: weight matrix incoming to layer *i* from layer *j*, *IW*_1,1_: weight matrix incoming to 1st hidden layer from input layer.

The number of neurons in input and output layers depends on the structure of the subject of interest.

An overall method for determining the number of hidden layers and neurons is not found in the literatures. Applying an evolutionary algorithm such the genetic algorithm (GA) and the particle swarm optimization (PSO) is one of the most widely used methods in this field.

### 2.3. The Basic Concept of Genetic Algorithm

GA is a part of evolutionary computing theory that is growing rapidly. The main idea of this algorithm lies in Darwin's theory of evolution. In every step of this method, an initial population of chromosomes (initial responses) produces a new population of chromosomes (secondary responses). Repeating this process and generating a new population from a previous one in each step result in population growth and optimum response.

The process of producing a new population from a previous one uses four operators as follows; Selection: This operator selects a number of chromosomes to produce the next generation in each population. The selected chromosomes are parents. The probability of selection of chromosomes with potential to reach more optimal results is higher than others. Crossover: This operator takes more than one parent solution and produces one or more child solutions from them. Mutation: Mutation is a genetic operator that alters one or more gene values in a chromosome from its initial state and will produce a new chromosome. Mutation helps to prevent the population from stagnating at any local optima. Elite: It is possible that some relatively optimal chromosomes are eliminated during the selection process so by this operator a number of elite chromosomes are transferred directly to the next population. 

**Figure 2:**
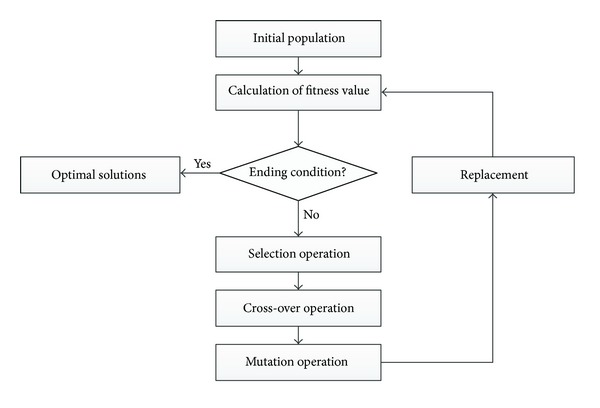
Genetic algorithm cycle.

Figure 2 demonstrates a general overview of the GA method.

### 2.4. Problem Definition

In this paper, the objective is to obtain a linear relation between the stressful life events introduced in the SLE questionnaire and the person's demographic characteristics. Demographic characteristics are considered as inputs and the stress level as an output variable. Stress level was measured by GHQ-12. In other words, we aim to determine the coefficients *α*_*i*_  (*i* = 1,2,…, 46) and *β*_*j*_  (*j* = 1,2, 3) in the following equation:

(2) y=α1x1+α2x2+⋯+αixi+⋯+α46x46+β1d1+β2d2+β3d3.

In the above equation, *x*_*i*_ is intensity of *i*, the stressful life event introduced in the SLE questionnaire. If the stressors actually exist in person's life, *x*_*i*_ is set to be the mean value of this stressful life event in the population; otherwise, in the case of lack of stressor in person's life, *x*_*i*_ is set to be equal to zero. Parameters *d*_1_, *d*_2_, and *d*_3_ are representing gender, age, and education level, respectively. Gender is a binary variable. Furthermore education level is a variable that is equal to educational years. *y* is the response variable determined by using the following formula:

(3) $$\begin{array}{c} y=\text{GHQ}\;\text{score}\times 25. \end{array}$$

If the GHQ score is less than 4, the individual is classified in the low stress level group and otherwise in the high stress level group. The cut-point score of 100 in GHO is well-defined. That means if *y* is less than 100, the subject is healthy otherwise is at risk of disease.

The purpose of this study is to measure the agreement between SLE questionnaire data and variable *y* as GHQ score changes. The SLE questionnaire data for a person is shown as follows:

(4) $$\begin{array}{c} \left(x_{1},x_{2},x_{3},\ldots ,x_{i},\ldots ,x_{46},d_{1},d_{2},d_{3}\right). \end{array}$$

Once we determined the coefficients, (2) was used as a screening tool.

### 2.5. Model Development

In this study, we have used a hybrid model of GA and ANN to obtain coefficients of variables (*α*_*i*_, *β*_*i*_) in (2). To extract the linear relationship between input data and response, variable ANN was used. In order to increase the efficiency of the ANN in this model, some of its effective parameters, such as the number of hidden neurons, are regulated by GA. Use of GA methods allows validation and regulation of values by searching in the domain and for number of hidden neurons. This is done in a way that the relation extracted by ANN affords the maximum discrimination and can discriminate healthy people from those at risk in the best way. 

After determining *α*_*i*_, in order to simplify the use of (2) as a screening tool, we changed this equation as follows:

(5) y=γ1x1′+γ2x2′+⋯+γixi′+⋯+γ46x46′+β1d1+β2d2+β3d3.

In the above equation, *γ*_*i*_ is product of *α*_*i*_ and *i* the stressor mean value. *x*_*i*_′ is a binary variable that is defined as follows:

(6) $$\begin{array}{c} x_{i}^{\prime }=\{\begin{array}{cc} 1, & \text{if}\;i\text{th}\;\text{stressor}\;\text{exist}\;\text{in}\;\text{individual},\\ 0, & \text{otherwise}. \end{array} \end{array}$$

We established an expert panel consisting of three psychiatrists, two psychologists, two epidemiologists, and one statistician to evaluate *γ*_*i*_ coefficients. At times, the expert panel changed some coefficients (*γ*_*i*_).

### 2.6. Sensitivity and Specificity

Once the coefficients in the equation were defined, the stress level of individuals was calculated (5). The resulting number was compared with the GHQ score. Sensitivity and specificity are calculated by defining the following variables: (a) true positive (TP) which means that individual diagnosis as sick person is truly correct, (b) true negative (TN) which means that healthy individuals are correctly diagnosed as healthy, (c) false positive (FP) which means that healthy individuals are incorrectly identified as, sick and (d) false negative (FN) which means that sick individuals are incorrectly diagnosed as healthy.

In our study, TP for an event is a calculated value greater than 100 and the GHQ score greater than 4. TN for an event is a calculated value less than 100 and the GHQ score of less than 4. FN for an event is a calculated value less than 100 and the GHQ score greater than 4. FP for an event is a calculated value of greater than 100 and the GHQ score less than 4. Sensitivity and specificity are calculated as follows:

(7) $$\begin{array}{c} \text{sensitivity}=\frac{\text{Number}\;\text{of}\;\text{TP}}{\text{Number}\;\text{of}\;\text{TP}+\text{Number}\;\text{of}\;\text{TN}},\\ \text{specificity}=\frac{\text{Number}\;\text{of}\;\text{TN}}{\text{Number}\;\text{of}\;\text{TN}+\text{Number}\;\text{of}\;\text{FP}}. \end{array}$$

## 3. Result

We studied 4569 individuals including a female population of 2252 (49.2%). The mean age of female and male participants was 38.6 ± 15.1 and 38.5 ± 15.4, respectively. The mean number of educational years in the female population was 8.9 ± 4.8 and in the male population was 7.2 ± 4.9 (mean ± standard deviation).

The SLE questionnaire included 46 stressors which were scored by a 6-point Likert scale (0 = never, 1 = very mild, 2 = mild, 3 = moderate, 4 = severe, and 5 = very severe). Mean scores for stressful life events in the study population are shown in Table 1.

As shown in Table 1, some financial stressors like financial inflation and low income have the greatest mean in the studied population. The higher the average score, the more the intensity of the stressor.

If zero was selected for specific stressors in the SLE questionnaire, the total score remains unchanged, and if any of the values from 1 to 5 was selected, the total score was replaced by the mean value of stressors. In addition to other stressors, a set of vectors was considered in some cases, including demographic characteristic and changed GHQ score. This set of vectors is used for training and testing neural networks.

After determining *α*_*i*_  (*i* = 1,2,…, 46) (as shown in (2)), the calculated coefficient for each stressor was multiplied by its stressor mean value. At the end, the resulting value was revised by the expert panel. We sorted the final results for weight of each stressor in order of their importance and impact in Table 2.

Among all stressful life events, death of parents, spouse, or siblings was the most important and influential in our studied population. In contrast, increased working hours and concern about addiction of a family member did not gain much scores and was not as important.

The results for the coefficients of demographic characteristics (*β*_1_ = 0, *β*_2_ = 0.2, *β*_3_ = 0) showed the effect of age on stress level for individuals and number of stressors. Changes in GHQ score increase, by 0.2 per year.

In this study, sensitivity was 83% and specificity was 81%. Our result shows that the SLE-R questionnaire is a screening tool with acceptable accuracy.

## 4. Discussion

In this paper, a revised SLE questionnaire is presented. We added weights to the SLE questionnaire's stressful life events using a hybrid model of GA and ANN. The importance of this promoted questionnaire is because of added weights to stressful life events. The weights calculated based on the population response. The SLE-R questionnaire is a well-established tool that differentiates individuals who are at risk of a health problem or disease from those who are not at risk. In this study sensitivity of 83% and specificity of 81% were obtained for a cut-point of 100.

Agreement between questionnaire data and criterion standards is the subject of many questionnaire-based studies. The objective of these studies is developing a diagnosis tool by determining a pattern between questionnaire data and a criterion standard. Many of these studies are dealt with multi-variables and it is highly likely that there are some interactions between them; ANNs are powerful tools that are used for correlations between known inputs and outputs and could consider the interaction between inputs in identified patterns.

Many studies have showed high efficiency of ANNs in various applications. DeGroff et al. used ANNs as a diagnostic tool and thereby classified heart sound data to innocent and pathological classes [19]. Sensitivity and specificity of 100% have been reported in their study. Wan et al. introduced a prediction approach based on a questionnaire using ANN models for skin attribute prediction [20]. The results of applying ANNs in the current study and other mentioned studies show that ANN is a strong and a valuable method.

The Holmes and Rahe stress scale (HRSS) is one of the most commonly and widely used quantitative measurements of psychosocial stress. It is a self-reported list of 43 common events associated with some degree of disruption of an individual's life. HRSS assists people to discern their internal stress and understand their cumulative stress over a one-year period. It assigns a number to the amount of stress being felt by a person with no margin for differential of how a person actually internalizes the stress. Individual's stress level is determined based on the calculated value in HRSS. User-friendliness is one of the prominent characteristics of HRSS that makes it to be commonly used. One of the other widely used stress scales named Life Experiences Survey (LES) that developed by Sarason et al. is a self-reported structured interview that assesses major life events in the past year. This user-friendly questionnaire includes 57 items divided into two sections. Each participants can record positive, negative, or no effect for each event on their life.

The SLE-R questionnaire that is proposed in this paper is a screening tool similar to HRSS and LES. Despite simplicity, these questionnaires are high-performance screening tools for investigating the stress levels. In many cases, clinical tests are costly and time consuming and associated with uncertainty. In this situation, clinical screening tools can help experts to better identify a diagnosis. Because these instruments are self-reported, individuals could gain some information about their own health status while filling them up.

In the SLE-R questionnaire, a global number is determined for each stressful life event, and differences between individuals are not considered. This questionnaire being self-reported is another limitation for our study because the participant could only remember that stressor that is subject of a question in the questionnaire. The SLE-R questionnaire has not been tested for normalization. The questionnaire should be compared with other questionnaires and tested in different communities.

In conclusion, the SLE-R questionnaire is introduced as a screening tool with high sensitivity and specificity. The features of this questionnaire make it a useful research tool that could be used in clinical and primary care settings. Offering clinical diagnostics to communities is very costly and not practical. Screening tools such as the SLE-R questionnaire are required to consider a smaller target group. The SLE-R questionnaire can be completed by individuals with low literacy.

## Figures and Tables

**Table 1:** Mean of stressful life events in studied population.

Domain	Stressful life events	Mean
	Addiction (self or family member)	0.18
	Divorce or separation	0.05
	Concern about addiction of a family members	0.45
Home life	Quarrels with spouse	0.31
	Being accused	0.29
	Legal problems	0.24
	Troubles with children	0.50

	Financial inflation	2.46
	Low income	2.06
Financial problems	Get into debt	1.69
	Taking on a mortgage	0.96
	Major financial problems	1.08

	Concern about your future	1.34
Social relations	Major social changes	0.77
	Social discrimination	1.15
	Social insecurity	1.01

	Loneliness	0.86
	Failure in achieving the life goals	1.04
Personal conflicts	Lack of social support	0.79
	Cultural alienation	0.65
	Not having an intimate friend	0.50

	Dealing with customers	0.50
Job conflicts	Quarrel with colleagues/boss	0.27
	Improper working place and environment	0.39
	Increased working hours	0.44

	Educational problems of children	0.43
Educational concerns	Participation major examinations	0.31
	Failure in major examinations	0.18
	High educational expenses	0.50

	Low salary	1.08
	High responsibility job	1.04
Job security	Concern about job future	1.00
	Job layoff	0.53
	Long-lasting unemployment	0.34

	Death of a close family member	0.81
Loss and separation	Major disease of family members leading to hospitalization	0.68
	Death of parents, spouse, or siblings	0.21
	Children's separation from family	0.29

	Birth of a child	0.21
Sexual life	Sexual relationship problems	0.12
	Pregnancy	0.08
	Unwanted pregnancy	0.05

Daily life	Major changes in sleeping and eating habits	0.71
	Air pollution and traffic	0.62

Health concerns	Mild illness	1.00
	Major physical disease leading to hospitalization	0.32

**Table 2:** Stressful life event score.

Stressful life events	Score
Death of parents, spouse, or siblings	46
Children's separation from family	32
Participation of major examinations	31
Low income	30
Long-lasting unemployment	29
Being accused	28
Educational problems of children	27
Major disease of family members leading to hospitalization	27
Concern about job future	26
Major financial problems	24
Unwanted pregnancy	22
Lack of social support	21
Failure in major examinations	21
Failure in achieving the life goals	19
Low salary	19
Major physical disease leading to hospitalization	19
High responsibility job	18
Concern about your future	17
Not having an intimate friend	17
Job layoff	16
Birth of a child	16
Addiction (self or family member)	15
Legal problems	14
Sexual relationship problems	14
Troubles with children	13
Quarrels with spouse	12
Mild illness	12
Divorce or separation	11
Financial inflation	11
Dealing with customers	11
Pregnancy	11
Major social changes	10
Death of close family member	10
Major changes in sleeping and eating habits	10
Social insecurity	9
Air pollution and traffic	7
Cultural alienation	6
Quarrel with colleagues/boss	6
Improper working place and environment	5
Get into debt	4
High educational expenses	4
Taking on a mortgage	3
Social discrimination	3
Loneliness	3
Concern about addiction of a family members	1
Increased working hours	1
